# Resveratrol promotes axonal regeneration after spinal cord injury through activating Wnt/β-catenin signaling pathway

**DOI:** 10.18632/aging.203628

**Published:** 2021-10-14

**Authors:** Zimin Xiang, Shuai Zhang, Xiaodong Yao, Libin Xu, Jianwei Hu, Chenghui Yin, Jianmei Chen, Hao Xu

**Affiliations:** 1 Department of Orthopaedics, The 900th Hospital, Joint Logistics Support Force, Fuzhou 350025, Fujian Province, P.R. China; 2 Department of Orthopaedics, Fuzong Clinical Medical College of Fujian Medical University, Fuzhou 350025, Fujian Province, P.R. China; 3 Department of Orthopaedics, Xiamen University Affiliated East Hospital Affiliated East Hospital, Fuzhou 350025, Fujian Province, P.R. China

**Keywords:** resveratrol, axonal regeneration, SCI, Wnt/β-catenin

## Abstract

Background: Spinal cord injury (SCI) is characterized by autonomic dysreflexia, chronic pain, sensory and motor deficits. Resveratrol has shown potential neuroprotective function in several neurodegenerative diseases’ models. However, if resveratrol could improve the function recovery after SCI and the further mechanism have not been investigated.

Methods: SCI rat model was established through laminectomy at lamina T9-10 aseptically. Basso, beattie and bresnahan (BBB) and inclined plane score, sensory recovery, spinal cord content, and inflammatory factors were measured. The levels of GAP43, NF421, GFAP, Bax, Bcl-2 and caspase-3 were measured using immunohistochemical staining. Tunel staining was applied to detect apoptosis level.

Results: Resveratrol significantly improved the function recovery, promoted axonal regeneration, suppressed apoptosis after SCI. The activation of Wnt/β-catenin signaling pathway was achieved by resveratrol. XAV939 significantly reversed the influence of resveratrol on function recovery, axonal regeneration, apoptosis after SCI.

Conclusions: Resveratrol could promote the function recovery and axonal regeneration, improve histological damage, inhibit apoptosis level after SCI through regulating Wnt/β-catenin signaling pathway. This research expanded the regulatory mechanism of resveratrol in SCI injury.

## INTRODUCTION

Spinal cord injury (SCI) refers to the spinal cord injury caused by external direct or indirect factors, which often leads to partial or total loss of sensory and motor functions below the injury level [1, 2]. Epidemiological investigation showed that the incidence of SCI was increasing year by year. Because SCI mostly occurs in young adults, and has a high disability rate, it is a huge burden to individuals and their families [3, 4]. Therefore, it is of great significance to promote the structural and functional reconstruction of injured spinal cord and improve the quality of life of patients. But so far, the treatment of SCI is still a difficult problem in the world. The current treatments of SCI include anti-inflammatory drugs, neuroprotective factors, cell transplantation and tissue engineering, but these treatments are not effective enough in nerve regeneration and functional recovery [2, 5, 6]. Therefore, it is necessary to explore the mechanism of axonal regeneration after neuron injury, and provide a new theoretical basis for the treatment of SCI.

Recent years, the function of resveratrol has been widely reported, and resveratrol is widely existed in grape, pine, Polygonum cuspidatum, cassia seed, peanut and other natural plants or fruits [7, 8]. Some reports have confirmed the preventive effect of resveratrol on cardiovascular disease and tumor. It has also been reported that resveratrol can promote the functional recovery of animals with spinal cord injury [9, 10], but the specific mechanism is still unclear.

Wnts containing wnt1, wnt3, and wnt5, are known as glycoproteins. Wnts have been proved to be closely related with cell proliferation, invasion, migration, and apoptosis in several pathological processes including tumor, myocardial ischemia reperfusion injury, and SCI [11–13]. Previous studies also confirmed the regulatory role of Wnt/β-catenin signaling pathway in the SCI process. It was reported that wnt3 could modulate the differentiation and proliferation of spinal cord neurons. The suppression of protein kinase inhibitors, stimulation of spinal cord precursor cells differentiation and axons regeneration, promotion of axons conduction, and inhibition of neuronal death could be also achieved through Wnt/β-catenin signaling pathway [14, 15]. However, if resveratrol could regulate the recovery after SCI through Wnt/β-catenin signaling pathway has not been investigated.

In this study, the SCI animal model was established to investigate the regulatory role of resveratrol in function recovery after SCI injury. We found that resveratrol could promote the function recovery and axonal regeneration, improve histological damage, inhibit apoptosis level after SCI through regulating Wnt/β-catenin signaling pathway. The novel findings of this research expand the regulatory mechanism of resveratrol in SCI injury, and might provide a potential therapeutic thought for SCI.

## MATERIALS AND METHODS

### SCI animal model

Sprague-Dawley rats (215-245 g, Vitalriver, Beijing, China) were used in this study. Animals were kept in the pathogen free animal center of the 900th hospital. The environment was maintained with 12 h light-dark cycle, 23-27° C. All experiment protocols have been approved by the experimental animal ethics committee of the 900th hospital, Joint Logistics Support Force (Approval number: 2020068). The animals were divided into the following groups randomly: Sham, SCI, SCI+resveratrol, SCI+resveratrol+XAV939. The animals in the sham group were treated with laminectomy. In the group SCI+resveratrol, 30 mg/kg of resveratrol was injected intraperitoneally daily for the first 14 days after SCI. In the group SCI+resveratrol+XAV939, 0.5 mg/kg of XAV939 and 30 mg/kg of resveratrol were injected intraperitoneally daily for the first 14 days after SCI [11, 16]. Same amount of saline was injected in the group sham and SCI.

Animals were anesthetized by intraperitoneal injection with xylazine (10 mg/kg) and ketamine (100 mg/kg) [17]. After anesthesia, the spinal cords of animals were exposed by laminectomy at lamina T9-10 aseptically. A 2 mm diameter impounder (10 g) was dropped from a height of 28 mm to strike the T9-10 spinal cord, leading to spinal cord congestion. The animals in the group sham only underwent laminectomy after anesthesia.

### Cell culture and treatment

Murine microglia BV2 cells were purchased from Chinese Academy of Science (Beijing, China). Cells were cultured in DMEM medium containing 10% fetal bovine serum (FBS, Gibco, USA) at 37° C with 5% CO_2_. After trypsin digestion, the cells were used for apoptosis measurement. The H_2_O_2_ treated cell model was established through culturing cell with H_2_O_2_ (100 μM) for 4 h at 37° C with 5% CO_2_. In the group H_2_O_2_+resveratrol, cells were incubated with resveratrol (10 μM) for 6 h after H_2_O_2_ treatment at 37° C with 5% CO_2_. In the group H_2_O_2_+resveratrol+XAV939, cells were incubated with resveratrol (10 μM) and XAV939 (5 μM) for 6 h after H_2_O_2_ treatment. In the group H_2_O_2_+resveratrol+iCRT3, cells were incubated with resveratrol (10 μM) and iCRT3 (5 μM) for 6 h after H_2_O_2_ treatment.

### CCK-8 assay

Cells (2×10^4^) were plated into the 96-well plate. The H_2_O_2_ treated cell model was established as described above. The cells in the group H_2_O_2_+resveratrol were cultivated with resveratrol (10 μM) for 6 h at 37° C with 5% CO_2_. The cells in the group H_2_O_2_+resveratrol+XAV939 were cultivated with resveratrol (10 μM) and XAV939 (5 μM) for 6 h at 37° C with 5% CO_2_. The cells in the group control were cultivated with same amount of PBS. Then, 20 μL CCK-8 regent was added to each well. After 1 h incubation, OD at 492 nm was measured.

### Tissue preparation

28 days after SCI, the animals were anesthetized using xylazine (10 mg/kg) and ketamine (100 mg/kg), and spinal cords were resected. The tissues were fixed using 4% paraformaldehyde for 48 h. Then, the tissues were dehydrated using 20% sucrose. The 10 μm crosswise sections were prepared for HE and immunohistochemical staining.

### Hematoxylin and eosin (HE) staining

The tissues were stained using hematoxylin for 45 s, and rinsed in distilled water for 10 s. HCl/95% alcohol (1:100) was used for differentiation (5 s). After washing using water for 30 min, tissues were stained with eosin for 15 s. Then, sections were washed with water for 10 min, and mounted using neutral gum after dehydration.

### Western blot

The spinal cord tissues were minced firstly, and homogenized using lysis buffer. After centrifugation (30 min at 4° C), the supernatant samples (30 μg) were subjected to SDS-PAGE, and transferred to a PVDF membrane (Sigma, USA). After blocking with TBST containing 10% non-fat milk for 1 h, the membrane was incubated with primary antibodies at 4° C for 12 h. The primary antibodies information was listed as follows. Wnt3a (1:1000, ab219412, Abcam, UK), Wnt2b (1:1000, ab178418, Abcam, UK), Wnt7a (1:1000, ab274321, Wnt5a (1:1000, ab179824, Abcam, UK), Abcam, UK), phospho-GSK-3β (1:1000, ab75814, Abcam, UK), β-catenin (1:1000, ab32572, Abcam, UK). The membrane was then incubated with secondary antibodies (Goat polyclonal Secondary Antibody to Rabbit IgG, 1:2000, ab150077, Abcam, UK) at 4° C for 2 h. Then, proteins were detected using an enhanced chemiluminescence detection kit (Sigma, USA), and protein bands was analyzed with ImageJ software.

To measure the protein expression of β-catenin in the nuclear, Nuclear Complex Co-IP Kit (Active Motif, Carlsbad, CA, USA) was used to exact nuclear firstly according to the instruction.

### qRT-PCR

Total RNA was extracted using the RNA extraction kit (Qiagen, Germany). ReverTra Ace qPCR RT Master Mix kit (Toyobo, Osaka, Japan) was used to synthesize cDNA. Then, ABI5500 system (Strata-gene) was use to perform qPCR. The reaction system was set as follows: 95° C (40 s), 40 cycles of 95° C (20 s), 60° C (40 s), 72° C (50 s). Primer sequences used listed as follows: Wnt3a (F: GGGACCCCAGTACTCCTCTC-30, R: GGGCATGATCTCCACGTAGT); β-catenin (F: TTCGCCTTCACTATGGACTACC, R: GCACGAACAAGCAACTGAACTA); GSK-3β (F: CGAUUACACGUCUAGUAUA, R: UAUACUAGACGUGUAAUCG); GAPDH (F: ACAACAGCCTCAAGATCATCAG, R: GGTCCACCACTGACACGTTG). The gene expression was analyzed using 2^-ΔΔCT^ method.

### Flow cytometry

Cells were firstly cultured at 37° C and 5% CO_2_. After different treatments as described in 2.2, the cells were digested using trypsin. After centrifugation for 5 min at 2500 rpm, the cells were collected and suspended with PBS. Then, propidium iodide (10 μL) and Annexin V-FITC (10 μL) were added to cells, and incubated for 20 min in the dark at room temperature. Finally, cell apoptosis was measured with flow cytometry.

### Immunohistochemical staining

The tissues were treated with 0.01 M citric acid for antigen retrieval. After washing with PBS, the slides were blocked with TBST containing 10 goat serum for 1 h. Then, the tissues were incubated with primary antibodies overnight at 4° C. The primary antibodies and dilution times were listed as follows. GAP43 (1:1000, ab232772, Abcam, UK), NF421 (1:800, ab187374, Abcam, UK), GFAP (1:1000, ab68428, Abcam, UK), Bax (1:800, ab81083, Abcam, UK), Cleaved Caspase-3 (1:1000, #961S, Cell signaling, USA), Bcl-2 (1:800, ab32124, Abcam, UK). Then, the sections were washed using PBS and incubated with related secondary antibodies for 2 h at room temperature. The secondary antibodies and dilution times were listed as follows. Goat anti-rabbit lgG (1:2000, ab205718, Abcam, UK) and Goat anti-mouse lgG (1:2000, ab205719, Abcam, UK). All sections were mounted and observed under a microscope (Leica, Germany). Image J software was used to quantify and normalize images.

### Tunnel staining

After antigen retrieval and blocking as described in the 2.9, the sections were incubated with primary antibodies overnight at 4° C. The sections were incubated with 15 μg/mL proteinase K (30 min), 0.2% hydrogen peroxide (40 min), and 0.1% Triton X-100 (5 min) successively. After washing with PBS, the tissues were blocked at 4° C for 4 h with 5% goat serum. After removing the serum, the sections were incubated with 5-bromo-4-chloro-3-indolyl phosphate (BCIP)/nitroblue tetrazolium (NBT) color liquid in the dark for 30 min at 37° C with 5% CO_2_. After washing with PBS, the sections were mounted and observed using a microscope.

### Basso, beattie and bresnahan (BBB) score and sensory recovery

The BBB score was measured as described previously [18]. The animals were put on a 2×3 m^2^ area for free movement. The behavior of hind limbs, tail, and trunk of animals were recorded. The BBB score was evaluated by three different experimenters who were blinded to the grouping. Von Frey filament test was applied to evaluate sensory recovery. Filaments with a gradient force were used to the paws to induce nociceptive responses (quick paw withdrawals from the stimuli). The withdrawal threshold value is defined as the minimal force inducing positive responses.

### Inclined plane test score

Animals were put on the inclined plane firstly. Then, the plane was then raised to the maximum angle, and the maximum vertical axis of the inclined plate was measured. Each rat was tested for 5 times.

### Inflammatory factors

The spinal cord tissues were minced firstly, and homogenized using lysis buffer. After centrifugation (30 min at 4° C), the supernatant samples were used to measure the expression of IL-6 (#PI328), IL-1β (#PI303), and TNF-*α* (#PT516) with related ELISA assay kits (Beyotime, China).

### Water content

The spinal cords were dried for 72 h at −80° C, and this was used to measure dry weight. Water content in spinal cords was calculated the formula: (wet weight − dry weight)/wet weight × 100%.

### Statistical analysis

Data was shown as mean ±SD, and analyzed with SPSS software (20.0, IBM, USA). An unpaired 2-tailed Student’s t-test was applied to compare the data of two groups. p <0.05 was believed to be statistically different.

### Data availability

Data supporting this study has been presented in the manuscript.

## RESULTS

### Resveratrol improved the function recovery after SCI

**Figure 1:**
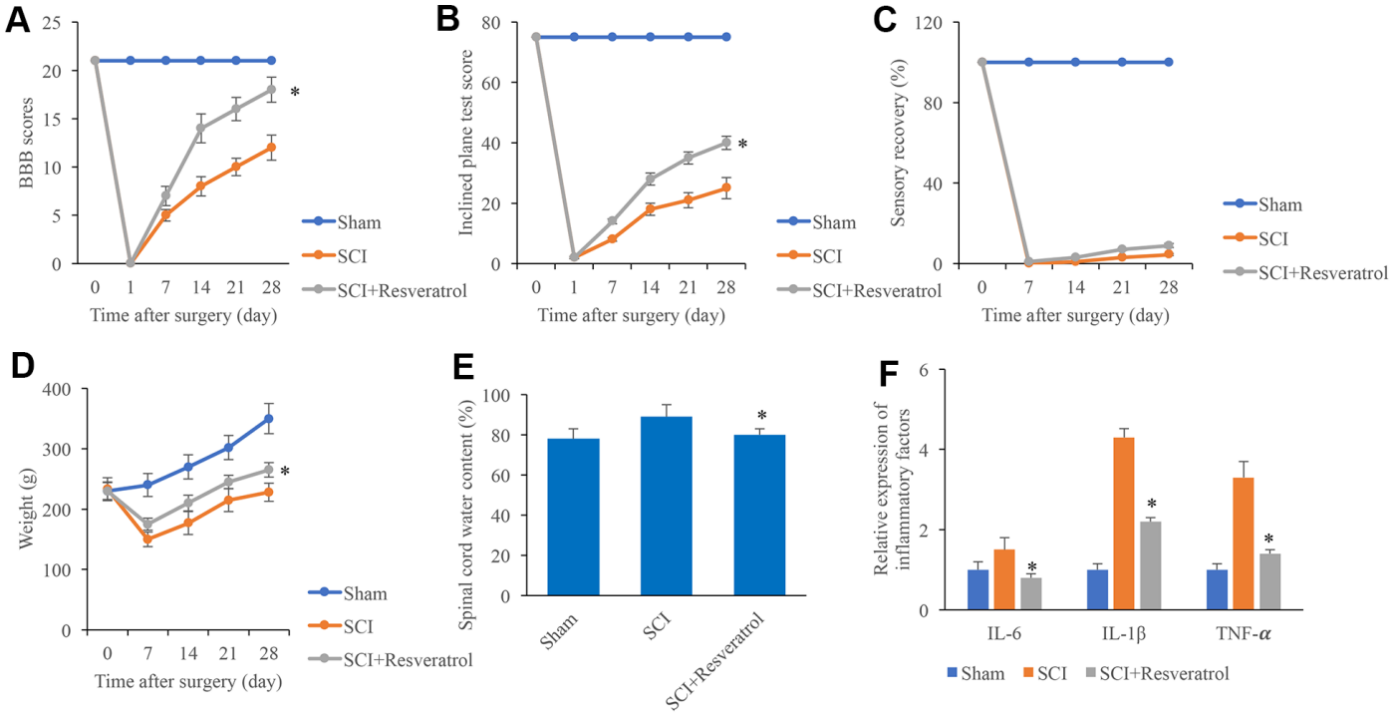
**Resveratrol improved the function recovery after SCI.** (**A**) The BBB scores were evaluated after operation at different time points; (**B**) The inclined plane test score was measured after operation at different time points; (**C**) The sensory recovery was measured after operation at different time points; (**D**) The weight of rats was measured after operation at different time points; (**E**) Spinal cord water content was detected; (**F**) Inflammatory factors were detected. *P <0.05 compared with group SCI. The experiments were repeated at least 3 independent times.

The BBB scores were evaluated after operation at different time points. The BBB scores dropped to zero 1 day after surgery in the group SCI and SCI+Resveratrol (Figure 1A), and it was increased gradually. However, the BBB scores in the group SCI+Resveratrol presented a remarkable faster increase compared with group SCI (Figure 1A). In addition, the rats in the group SCI+Resveratrol presented a significant faster recovery in terms of inclined plane score (Figure 1B) and weight (Figure 1D). No significant difference was observed in sensory recovery between group SCI+Resveratrol and SCI (Figure 1C). Lower spinal cord water content (Figure 1E) and inflammatory factors (Figure 1F) including IL-6, IL-1β, and TNF-α were found in the group SCI+Resveratrol compared with group SCI.

### Resveratrol promoted axonal regeneration after SCI

**Figure 2:**
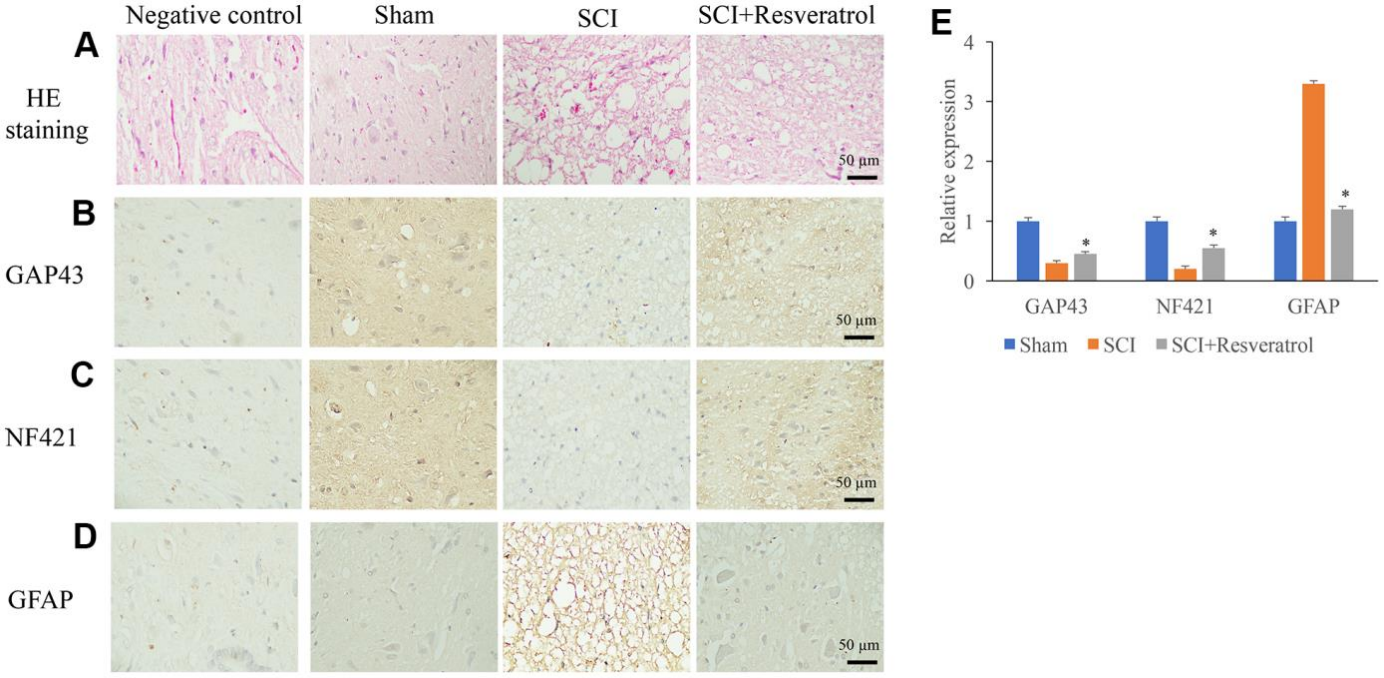
**Resveratrol promoted axonal regeneration after SCI.** (**A**) Histological changes were evaluated using HE staining; (**B**) The expression of GAP43 was measured using IHC staining; (**C**) The expression of NF421 was measured using IHC staining; (**D**) The expression of GFAP was measured using IHC staining; (**E**) The levels of GAP43, NF421, and GFAP were analyzed. *P <0.05 compared with group SCI. The experiments were repeated at least 3 independent times.

Histological changes were evaluated using HE staining. In the group sham, clear and symmetric axons, myelin, nerve fibers could be observed. Nerve cells could be observed in the gray matter (Figure 2A and Supplementary Figure 1). However, in the group SCI, a large number of inflammatory cells were observed in the tissues (Figure 2A). Meanwhile, deformation of spinal cord and large spaces between tissues were found. The treatment with resveratrol significantly reversed the histological changes after SCI. In addition, the expression of axonal regeneration and nerve cell regeneration markers, GAP43 and NF421, were detected. After SCI administration, the expression of GAP43 and NF421 were remarkably inhibited (Figure 2B, 2C, 2E). However, resveratrol markedly increased the levels of GAP43 and NF421 compared with group SCI. The marker of astrocytes, GFAP, was also measured. Remarkable increase of GFAP was found in the group SCI compared with group sham and SCI+resveratrol (Figure 2D, 2E).

### Resveratrol suppressed apoptosis *in vivo* and *in vitro*

**Figure 3:**
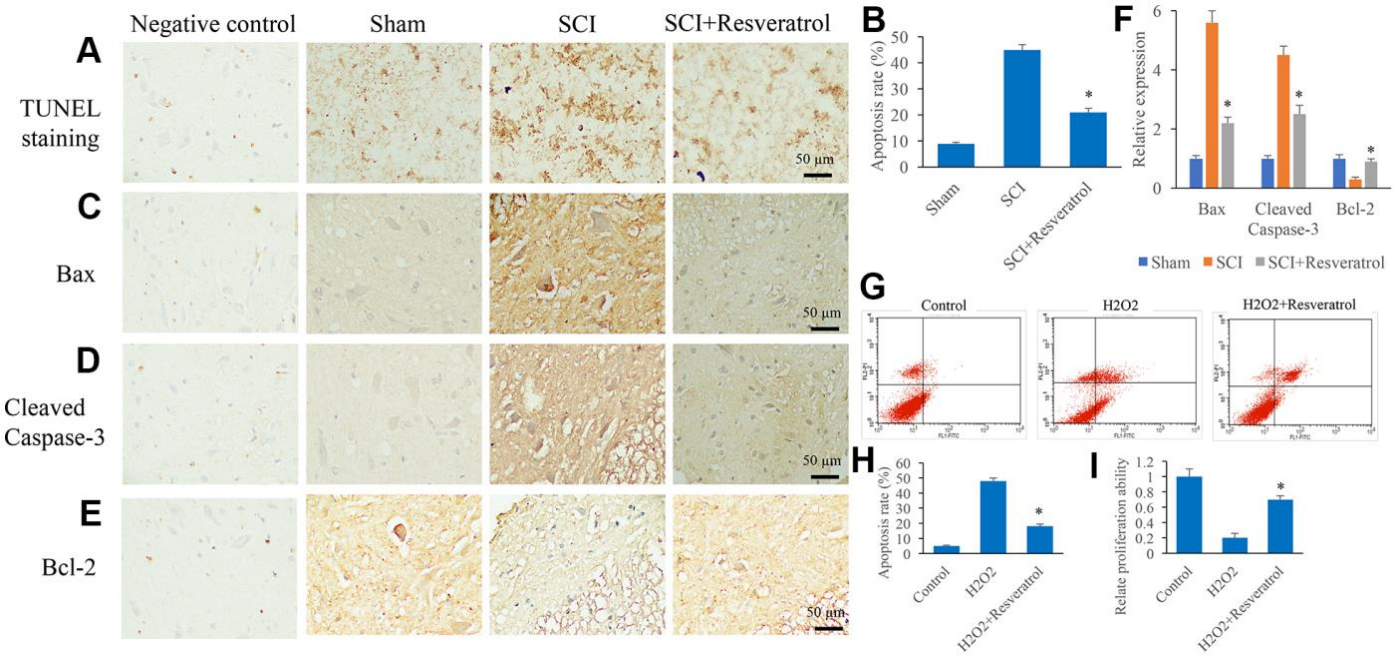
**Resveratrol suppressed apoptosis *in vivo* and *in vitro*.** (**A**) The apoptosis level in the tissue was detected using TUNEL staining; (**B**) The apoptosis level in the tissue was analyzed; (**C**) The expression of Bax was measured in the tissues; (**D**): The expression of Cleaved Caspase-3 was measured in the tissues; (**E**): The expression of Bcl-2 was measured in the tissues; (**F**): The levels of Bax, Cleaved Caspase-3, and Bcl-2 was analyzed; (**G**): The cell apoptosis model was established by H_2_O_2_; (**H**): The remarkable increased apoptosis level induced by H_2_O_2_ was suppressed by resveratrol; (**I**) The decreased cell proliferation ability caused by H_2_O_2_ was promoted by resveratrol. *P <0.05 compared with group SCI or H_2_O_2_. The experiments were repeated at least 3 independent times.

The apoptosis level in the tissue was detected using TUNEL staining. The apoptosis level in the group SCI was remarkably elevated compared group sham, but it was significantly suppressed after resveratrol treatment (Figure 3A, 3B and Supplementary Figure 2). Meanwhile, pro-apoptosis proteins, Bax and Cleaved Caspase-3, were remarkably increased, but anti-apoptosis protein, Bcl-2, was inhibited in the group SCI compared with group sham (Figure 3C–3F). However, the influence of SCI on the expression of Bax, Cleaved Caspase-3, and Bcl-2 was remarkably reversed by resveratrol (Figure 3C–3F). The influence of resveratrol on apoptosis was also evaluated *in vitro*. H_2_O_2_ was used to establish cell apoptosis model *in vitro*. The remarkable increased apoptosis level induced by H_2_O_2_ was suppressed by resveratrol (Figure 3G, 3H). Meanwhile, decreased cell proliferation ability caused by H_2_O_2_ was promoted by resveratrol (Figure 3I).

### Resveratrol activated the Wnt/β-catenin signaling pathway *in vivo*

**Figure 4:**
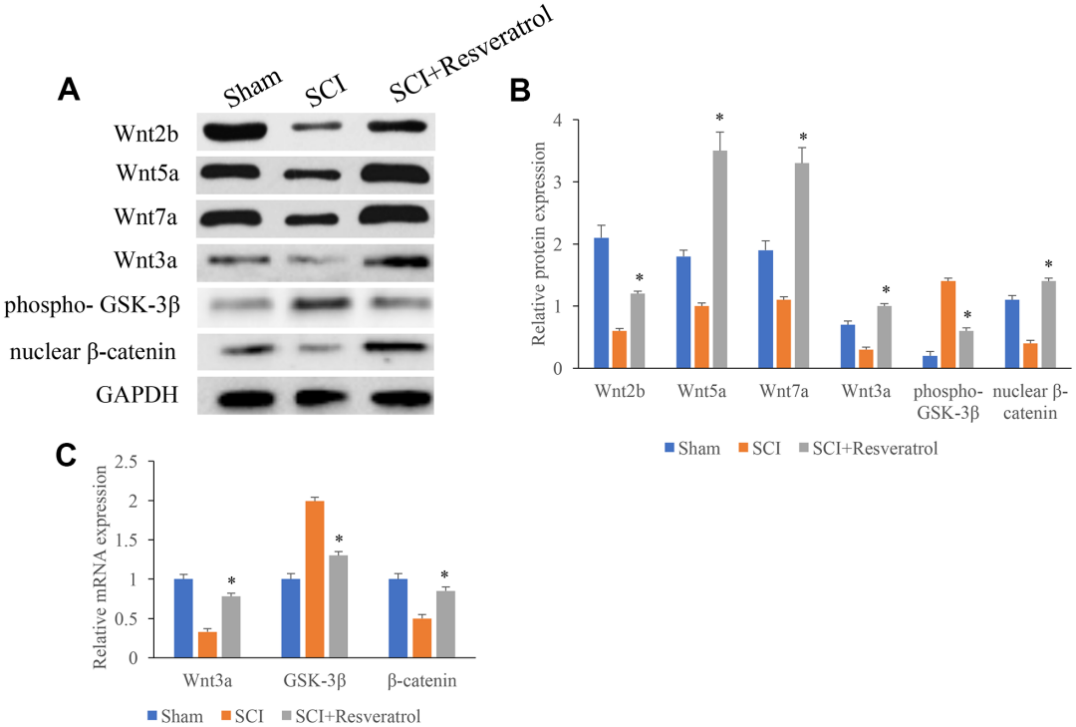
**The protein and mRNA expression of Wnt3a, GSK-3β, and β-catenin in the tissues were measured.** (**A**) The protein expression of Wnt3a, GSK-3β, and β-catenin in the tissues were measured using western blotting; (**B**) The protein expression of Wnt3a, GSK-3β, and β-catenin in the tissues were measured analyzed; (**C**) The mRNA expression of Wnt3a, GSK-3β, and β-catenin in the tissues were measured using qRT-PCR. *P <0.05 compared with group SCI. The experiments were repeated at least 3 independent times.

The protein and mRNA expression of Wnt3a, GSK-3β, and β-catenin in the tissues were measured. The protein and mRNA levels of Wnt3a and β-catenin were significantly inhibited after SCI treatment (Figure 4A–4C) compared with group Sham. However, resveratrol remarkably reversed the influence of SCI, and increased the levels of Wnt3a and β-catenin, but decreased the expression of GSK-3β (Figure 4A–4C).

### XAV939 significantly reversed the influence of resveratrol on function recovery after SCI

**Figure 5:**
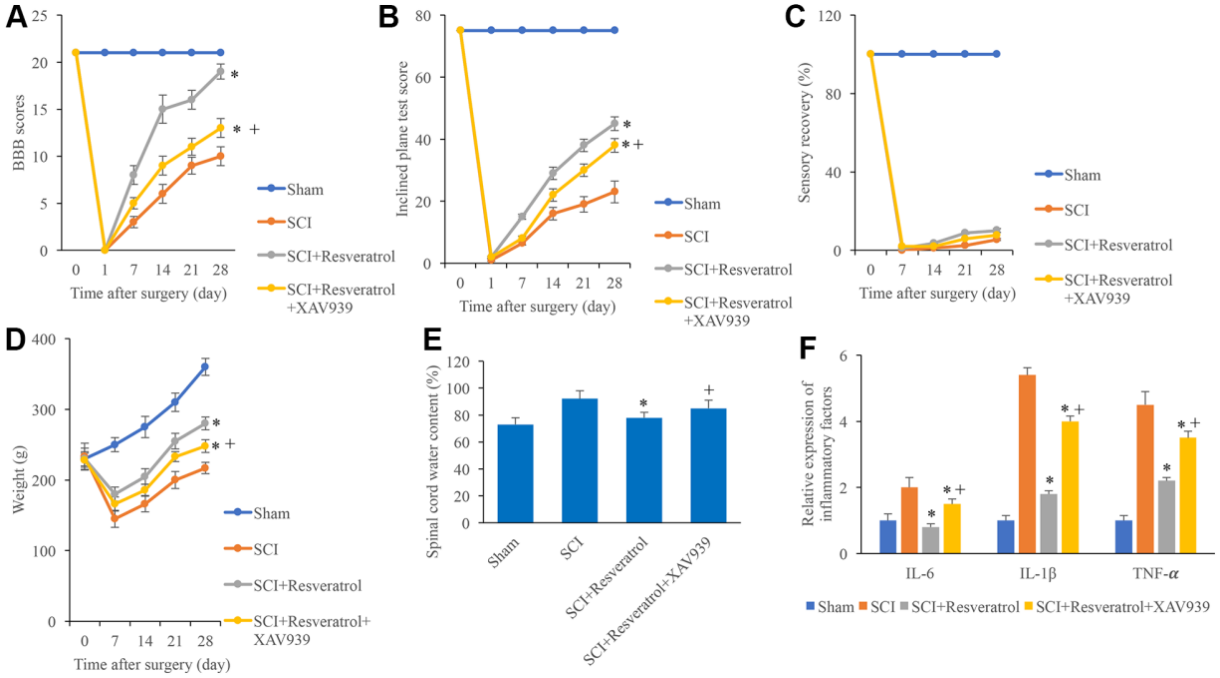
**XAV939 significantly reversed the influence of resveratrol on function recovery after SCI.** (**A**) The BBB scores were evaluated after operation at different time points; (**B**) The inclined plane test score was measured after operation at different time points; (**C**) The sensory recovery was measured after operation at different time points; (**D**) The weight of rats was measured after operation at different time points; (**E**) Spinal cord water content was detected; (**F**) Inflammatory factors were detected. *P <0.05 compared with group SCI, + P <0.05 compared with group SCI+resveratrol. The experiments were repeated at least 3 independent times.

XAV939, the inhibitor of Wnt/β-catenin signaling pathway, was used to investigate the regulation of resveratrol on Wnt/β-catenin signaling pathway. Significant higher levels of BBB score, inclined plane score, sensory recovery, and weight gaining caused by resveratrol were markedly decreased by XAV939 (Figure 5A–5D). In addition, the decreased spinal cord water content (Figure 5E) and inflammatory factors (Figure 5F) caused by resveratrol were remarkably promoted by XAV939 suggesting that resveratrol might improve function recovery after SCI via Wnt/β-catenin signaling pathway.

### XAV939 significantly reversed the influence of resveratrol on axonal regeneration after SCI

**Figure 6:**
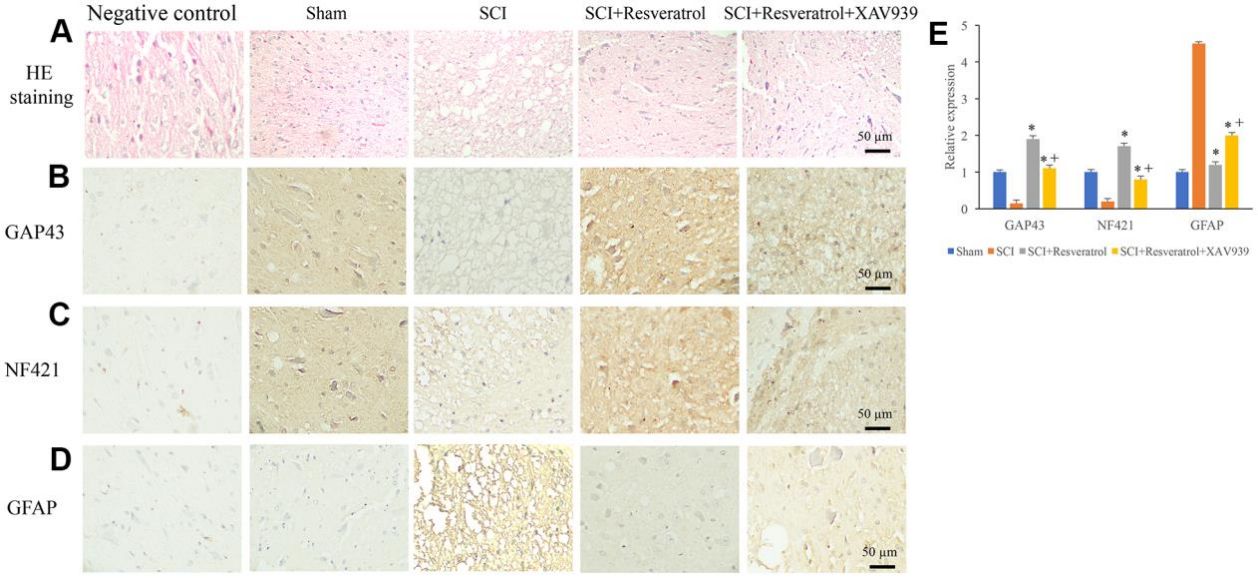
**XAV939 significantly reversed the influence of resveratrol on axonal regeneration after SCI.** (**A**) Histological changes were evaluated using HE staining; (**B**) The expression of GAP43 was measured using IHC staining; (**C**) The expression of NF421 was measured using IHC staining; (**D**) The expression of GFAP was measured using IHC staining; (**E**) The levels of GAP43, NF421, and GFAP were analyzed. *P <0.05 compared with group SCI, + P <0.05 compared with group SCI+resveratrol. The experiments were repeated at least 3 independent times.

We found that the influence of SCI on histological changes, the expression of GAP43, NF421, and GFAP could be remarkably reversed by resveratrol (Figure 6A–6E and Supplementary Figure 3). However, after additional treatment with XAV939, the influence of resveratrol on histological changes, the expression of GAP43, NF421, and GFAP were significantly reversed (Figure 6A–6E). These findings indicated that resveratrol might regulate axonal regeneration after SCI through targeting Wnt/β-catenin signaling pathway.

### XAV939 significantly reversed the influence of resveratrol on apoptosis after SCI

**Figure 7:**
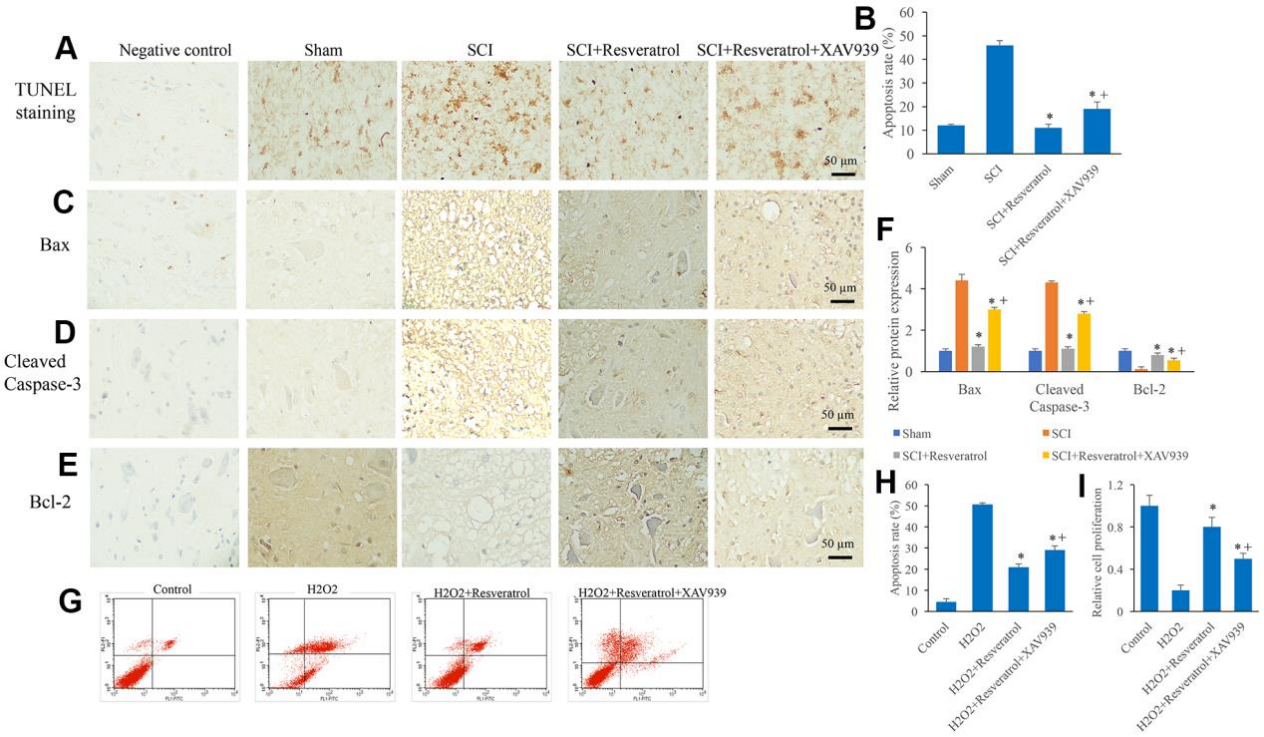
**XAV939 significantly reversed the influence of resveratrol on apoptosis after SCI.** (**A**) The apoptosis level in the tissue was detected using TUNEL staining; (**B**) The apoptosis level in the tissue was analyzed; (**C**) The expression of Bax was measured in the tissues; (**D**): The expression of Cleaved Caspase-3 was measured in the tissues; (**E**): The expression of Bcl-2 was measured in the tissues; (**F**): The levels of Bax, Cleaved Caspase-3, and Bcl-2 was analyzed; (**G**): The cell apoptosis model was established by H_2_O_2_; (**H**): The remarkable increased apoptosis level induced by H_2_O_2_ was suppressed by resveratrol; (**I**) The decreased cell proliferation ability caused by H_2_O_2_ was promoted by resveratrol. *P <0.05 compared with group SCI or H_2_O_2_, + P <0.05 compared with group SCI+resveratrol. The experiments were repeated at least 3 independent times.

Similar to previous findings, the apoptosis in the tissues (Figure 7A, 7B and Supplementary Figure 4) and cells (Figure 7G, 7H) were remarkably suppressed in the group SCI+resveratrol compared with group SCI. Meanwhile, the increased expression of Bax (Figure 7C, 7F) and cleaved caspase-3 (Figure 7D, 7F), and decreased expression of Bcl-2 (Figure 7E, 7F) caused by SCI were also reversed in the group SCI+resveratrol. However, simultaneous treatment with XAV939 and resveratrol markedly reversed the influence of resveratrol on apoptosis and related proteins expression (Figure 7A–7H). In addition, the cell proliferation was significantly suppressed in the group SCI+resveratrol+XAV939 compared with group SCI+resveratrol (Figure 7I). These data suggested that resveratrol might suppress apoptosis after SCI through Wnt/β-catenin signaling pathway.

### iCRT3 significantly reversed the influence of resveratrol on apoptosis *in vitro*

**Figure 8:**
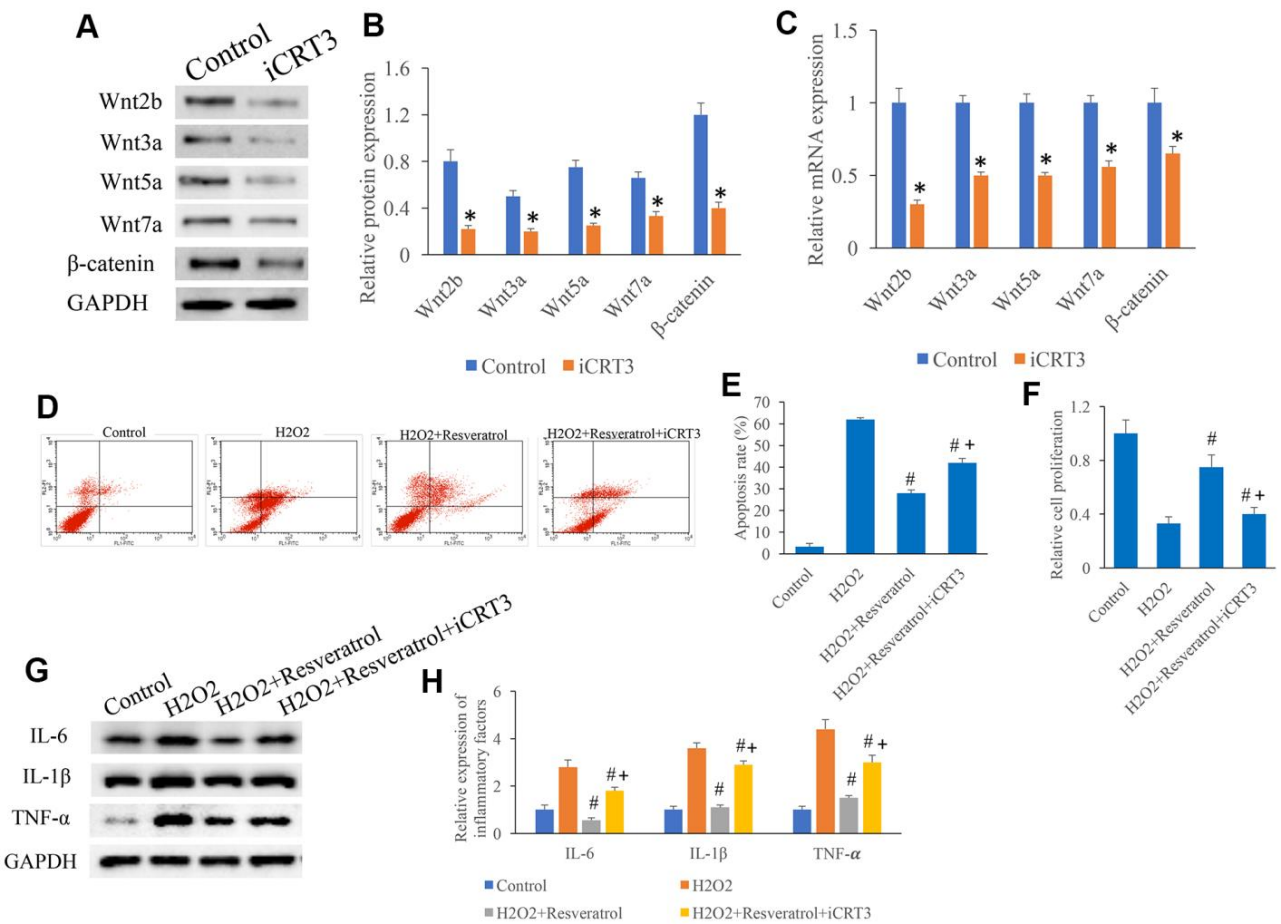
**iCRT3 significantly reversed the influence of resveratrol on apoptosis *in vitro*.** (**A**) Influence of iCRT3 on the Wnt signaling related proteins expression through western blotting; (**B**) CRT3 significantly inhibited the protein expression of Wnt signaling related proteins; (**C**) iCRT3 significantly inhibited the mRNA expression of Wnt signaling related proteins; (**D**) The influence of iCRT3 on the apoptosis was investigated; (**E**) iCRT3 significantly reversed the influence of resveratrol on apoptosis *in vitro*; (**F**) iCRT3 significantly reversed the influence of resveratrol on cell proliferation *in vitro*; (**G**) Influence of iCRT3 on the protein expression of inflammatory factors through western blotting; (**H**) iCRT3 significantly reversed the influence of resveratrol on inflammatory factors expression. *P <0.05 compared with group control, #P <0.05 compared with group H_2_O_2_, + P <0.05 compared with group SCI+resveratrol. The experiments were repeated at least 3 independent times.

Another inhibitor of Wnt/β-catenin signaling, iCRT, was used to investigate the role of Wnt/β-catenin signaling in apoptosis *in vitro*. iCRT remarkably suppressed the protein (Figure 8A, 8B) and mRNA (Figure 8C) levels of Wnt2b, Wnt3a, Wnt5a, Wnt7a, and β-catenin. The inhibition effect of resveratrol on apoptosis (Figure 8D, 8E) and cell proliferation (Figure 8F) were reversed by iCRT. Meanwhile, the suppression of resveratrol on inflammatory factors was promoted by iCRT (Figure 8G, 8H).

## DISCUSSION

SCI always causes irreversible physical function injury, and it is still a global challenge. The current treatment drug of SCI, methylprednisolone, has been proved to exert some complications such as pulmonary embolism and infection [19, 20]. The slight neuroprotective effect by methylprednisolone also limit its further application in SCI injury.

The difficulty of SCI treatment is to promote the injured axons to regenerate and link with target cells, so as to achieve nerve reinnervation and functional recovery [21]. Axon regeneration process includes axon sprouting, growth, extension, and reconnection of axon with target cells to achieve nerve reinnervation and function recovery [22]. Different from peripheral nerves, axons of adult mammalian central nervous system often cannot regenerate effectively after injury [23]. The reasons that affect axon regeneration are complex. The main factors affecting axon regeneration after SCI can be summarized as follows: (1) Influence of inhibitors on local microenvironment of damage; (2) Mechanical disorder of glial scar; (3) The injured neurons of the central nervous system have inherent defects in the ability of regeneration [24, 25].

Resveratrol has been proved to be beneficial in a broad type of pathological conditions including tumor, myocardial ischemia reperfusion injury, and oxidative stress [16]. Previous study indicated that resveratrol protected against SCI via activating autophagy and inhibiting apoptosis through SIRT1/AMPK [26]. Meanwhile, resveratrol could improve neurological outcome and neuroinflammation after SCI through enhancing autophagy via AMPK/mTOR and Notch [27, 28]. In addition, resveratrol protected SCI from hypoxic injury by activating Nrf-2 [29]. In this study, we demonstrated that resveratrol promoted axonal regeneration after SCI through activating Wnt/β-catenin signaling pathway, which expand the function mechanism how resveratrol affects SCI. The neuroprotective effects of resveratrol in the fields of central nervous system diseases such as traumatic brain injury, subarachnoid hemorrhage, and stroke were also confirmed [26]. It was reported that resveratrol could improve the neurological recovery and decrease inflammation after SCI through promoting autophagy via AMPK/mTOR pathway [27]. In addition, the inflammatory factors caused by SCI were alleviated by resveratrol via increasing SIRT1 and inhibiting NF-κB [30]. In this study, we found that resveratrol could significantly activate the levels of Wnt3a and β-catenin, suppress GSK-3β (Figure 4). Meanwhile, the inhibitor of Wnt/β-catenin signaling pathway, XAV939, markedly reverse the influence of resveratrol on neurological recovery, axonal regeneration, and apoptosis after SCI. These findings indicated that resveratrol might regulate the recovery process after SCI through Wnt/β-catenin signaling pathway.

GAP43, also known as neurodulin, is an axon membrane protein. It is a nerve specific protein, which is involved in nerve extracellular growth, synaptic development and regeneration. GAP-43 is highly expressed during neuronal development and regeneration. It can mediate axon extension [31]. NF421 is a kind of neurofilament heavy polypeptide. Both GAP43 and NF421 could be viewed as the markers of nerve regeneration. GFAP is a marker of astrocyte activation [32]. The expression regulation of GAP43, NF421, and GFAP should be the potential regulatory mechanism how resveratrol improves neurological function after SCI injury.

Apoptosis is closely related with the process of tissue destruction and cell stress, and the level of apoptosis is highly elevated after SCI. Apoptosis could cause nerve impairment and further induce sensory and motor damage [33]. In addition, apoptosis acts an important role during neurons death and spinal cord microcirculatory damage after SCI [34]. It was reported that metformin could reduce inflammation and apoptosis, and promote functional recovery of SCI rats through activating Wnt/β-catenin [11]. Wnt-3a could improve functional recovery through autophagy activation via inhibiting the mTOR signaling pathway after SCI [35]. In this study, resveratrol remarkably decreased the apoptosis level after SCI. However, the inhibitor of Wnt/β-catenin signaling pathway, XAV939, significantly reversed the influence of resveratrol indicating that resveratrol might regulate the apoptosis process through targeting Wnt/β-catenin signaling pathway. This is might be one potential regulatory mechanism how resveratrol improves function recovery after SCI injury. However, there are some limitations in this study. Firstly, the further mechanism how resveratrol affects axonal regeneration is not fully clarified. Secondly, the dose-response experiments were not performed in this study, and the doses of resveratrol and XAV939 might not be the best. Further study needs to be conducted to find a better dose.

## CONCLUSIONS

In this study, we demonstrated that resveratrol could significantly improve neurological function recovery, promote axonal regeneration, and suppress apoptosis after SCI injury through activating Wnt/β-catenin signaling pathway. This study unfolds the novel regulatory mechanism of resveratrol in improving neurological recovery. Meanwhile, this study might provide a potential therapeutic strategy for the treatment of SCI through resveratrol promoting axonal regeneration and activating Wnt/β-catenin signaling pathway.

## Supplementary Material

Supplementary Figures
