# Supplementary material for: Resveratrol promotes axonal regeneration after spinal cord injury through activating Wnt/β-catenin signaling pathway
Source: Aging (Albany NY). 2021 Oct 14;13(20):23603–19. doi: 10.18632/aging.203628 (PMC8580349; doi:10.18632/aging.203628)
Supplement: Supplementary Figures [file aging-13-203628-s001.pdf]

SUPPLEMENTARY FIGURES

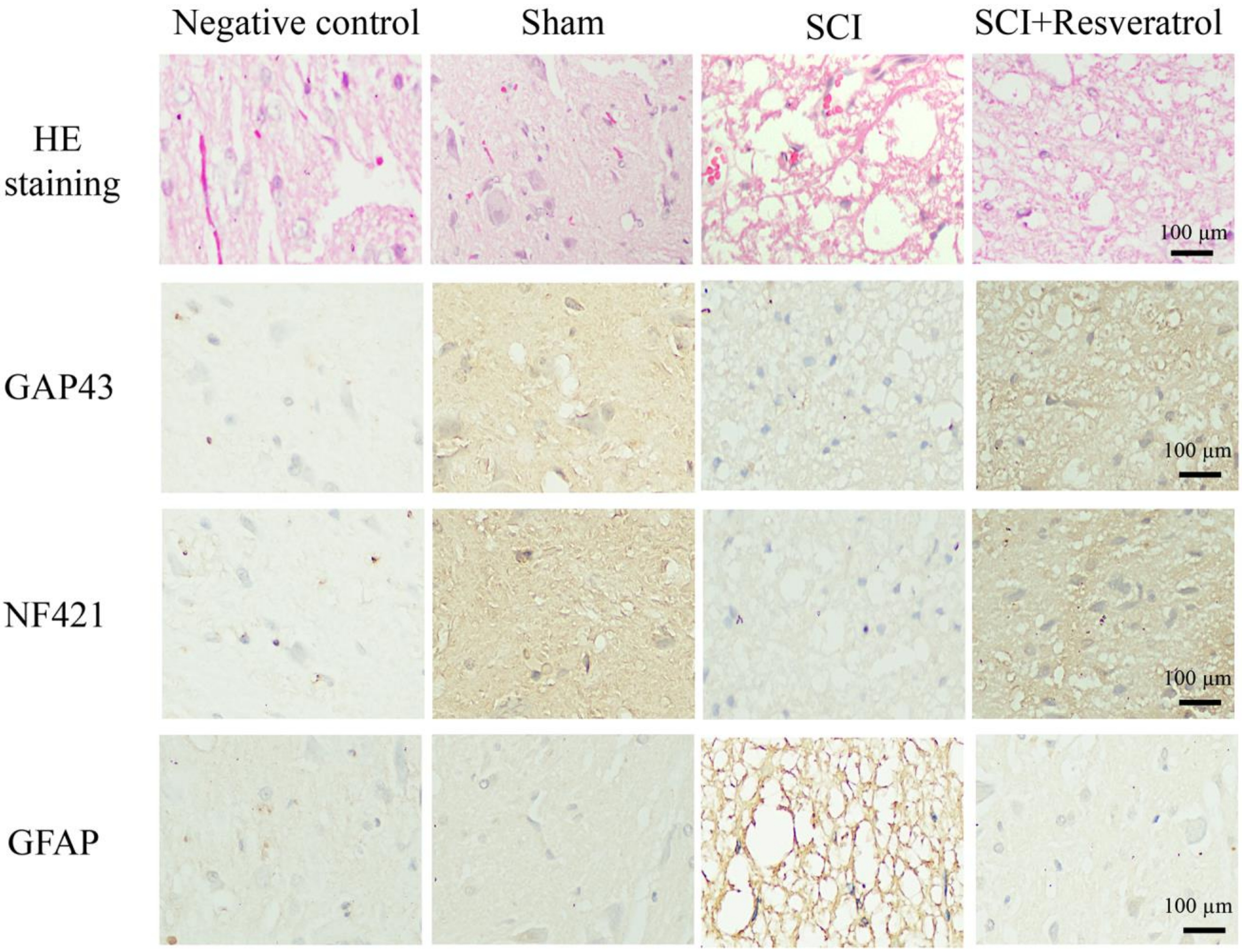

Supplementary Figure 1. Higher magnification histological images indicated the promotion of axonal regeneration by resveratrol after SCI.

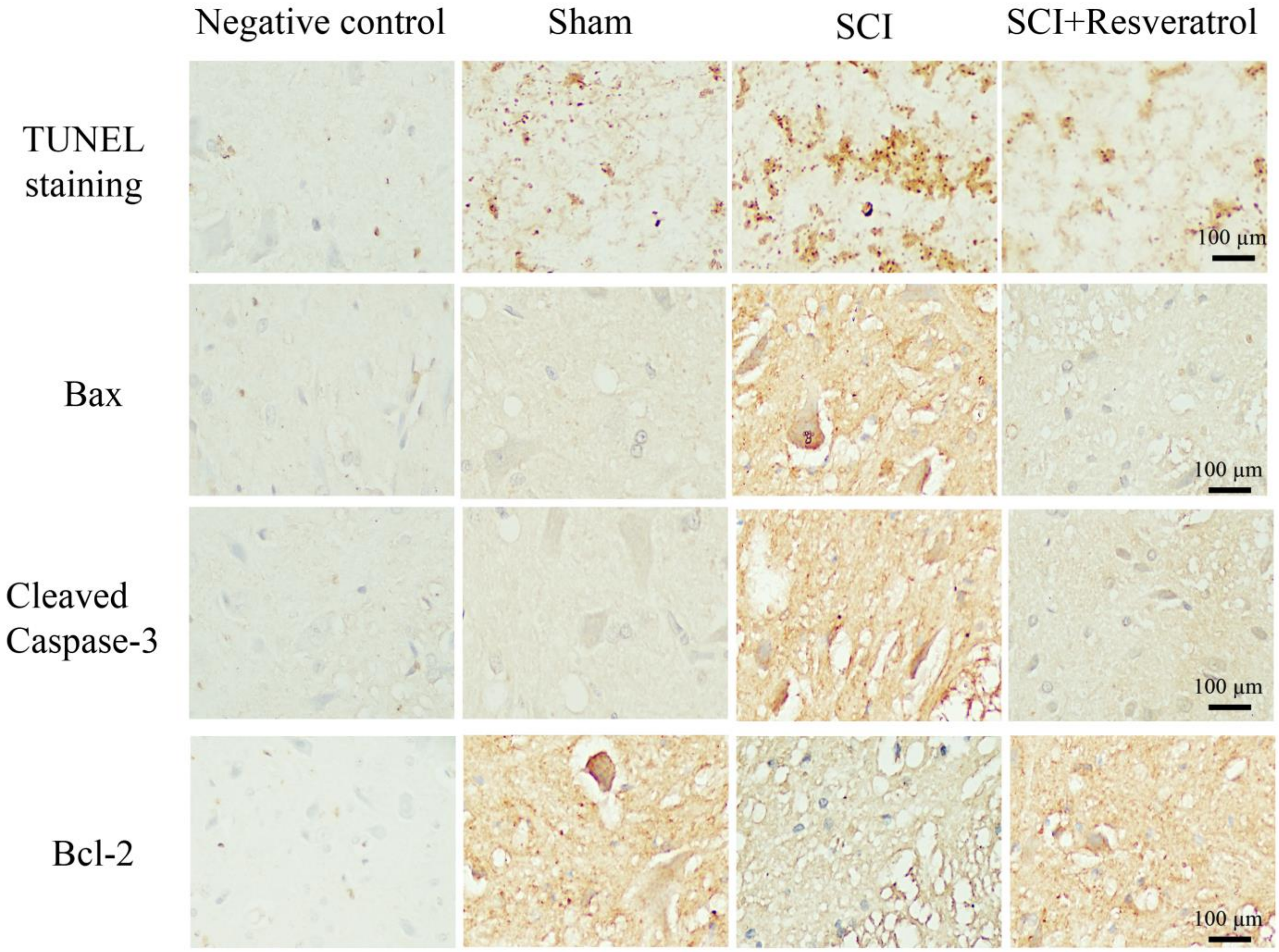

Supplementary Figure 2. Higher magnification histological images indicated the inhibition of apoptosis by resveratrol after SCI.

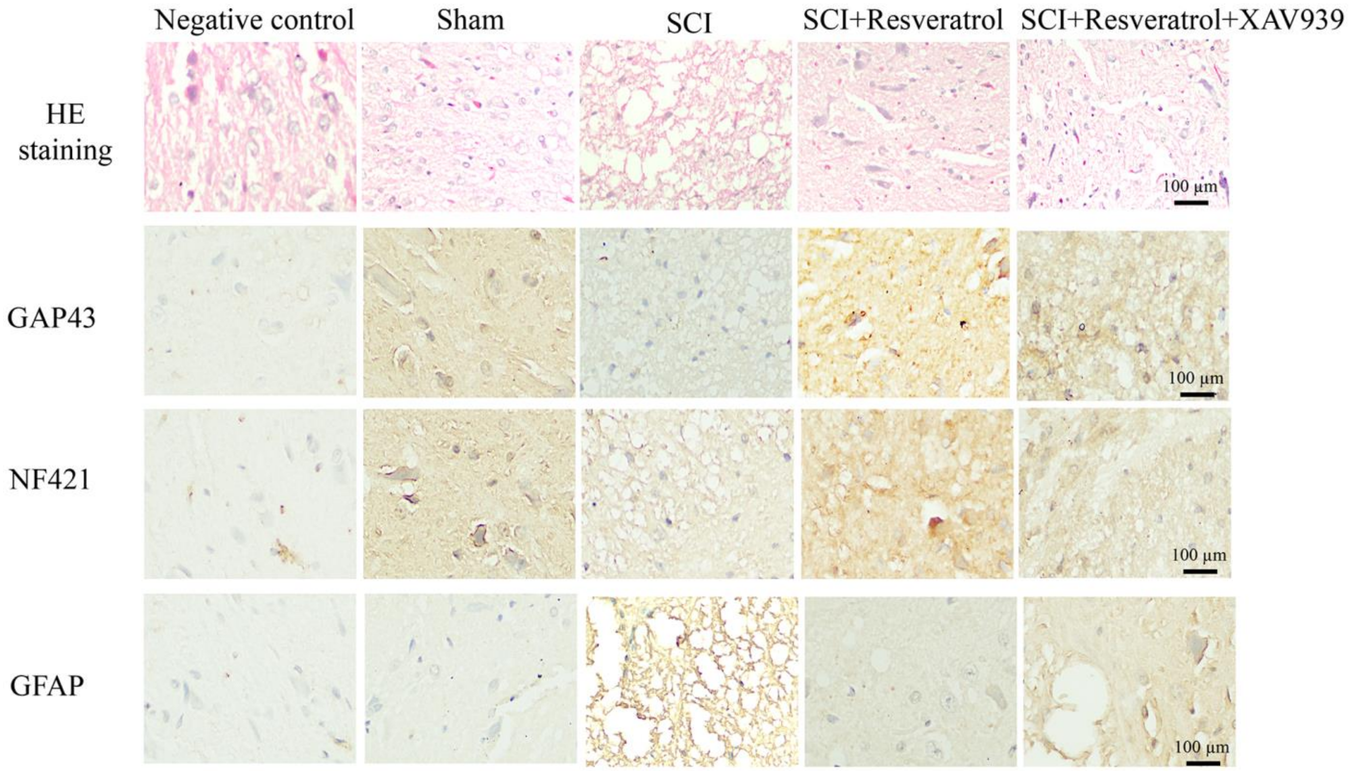

Supplementary Figure 3. Higher magnification histological images indicated the revised effect of XAV939 on the influence of resveratrol on axonal regeneration after SCI.

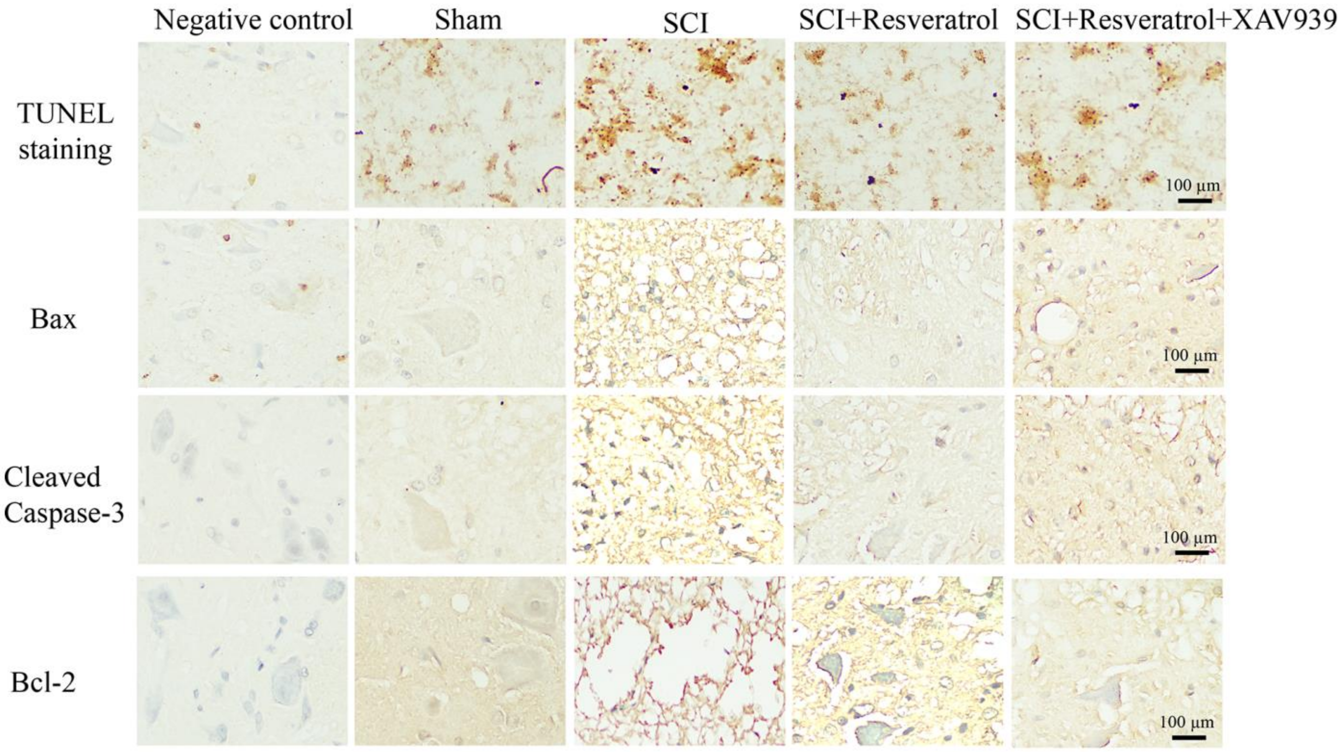

Supplementary Figure 4. Higher magnification histological images indicated the revised effect of XAV939 on the influence of resveratrol on apoptosis after SCI.
